# 614. Evaluating the Use of Dalbavancin for Off-Label Indications

**DOI:** 10.1093/ofid/ofab466.812

**Published:** 2021-12-04

**Authors:** Katherine Taylor, John Williamson, Tyler Stone, James Johnson, Zachary Gruss, Vera Luther, Vera Luther, Courtney Russ-Friedman, Chris Ohl, James Beardsley

**Affiliations:** 1 Wake Forest Baptist Health System, Winston Salem, NC; 2 Wake Forest School of Medicine, Winston Salem, NC

## Abstract

**Background:**

Dalbavancin (dalba) is a long-acting antibiotic (ABX) approved for skin and soft tissue infections. Post-marketing data suggests dalba is being used for off-label indications that require long term IV ABX; however, data assessing this off-label usage is limited. The purpose of this study was to evaluate the real-world efficacy, safety, and financial impact of off-label dalba use.

**Methods:**

Setting: 4-hospital health system. Design: retrospective, observational study. Adult patients (pts) who received dalba from Jan 2018 to Jan 2021 for an off-label indication were included. Pts who were pregnant or had an infection caused by a pathogen outside dalba’s antimicrobial spectrum were excluded. Primary outcome was clinical success at 90 days defined as no need for additional ABX (excluding suppression therapy) or surgical intervention following dalba therapy and no positive cultures post treatment associated with the dalba-targeted infection. Secondary outcomes included safety (nephrotoxicity and hepatotoxicity). A financial analysis was performed by subtracting the cost of dalba from the anticipated cost of pt stay [&427/day for hospital; &262/day for skilled nursing facility (SNF)] if standard IV therapy was given.

**Results:**

50 pts met study criteria; 42% were IV drug users; 14% were self-pay. Indications included osteomyelitis (54%), endocarditis (22%), bacteremia (16%), and prosthetic joint infection (PJI) (8%). The predominant organism was *S. aureus* (60%), with 42% caused by MRSA. All but 1 pt received 1.5 g of dalba. 20 (40%) pts received 1 dose; 26 (52%) received 2. Overall, 43 (86%) pts achieved clinical success at 90 days, including 87% of osteomyelitis/PJI pts, 82% of endocarditis pts, and 100% of pts with bacteremia. There were no instances of nephrotoxicity or hepatotoxicity. Estimated cost avoidance per pt was &5210 and &1652 if traditional IV therapy was completed in the hospital and SNF, respectively. Because the alternative therapy to dalba could not be predicted, these costs were not included in analysis but likely would have increased calculated cost avoidance.

**Conclusion:**

Dalba was associated with a relatively high success rate for the treatment of off-label indications and may have less total costs than traditional IV ABX.

**Disclosures:**

**James Johnson, PharmD**, **FLGT** (Shareholder) **Vera Luther, MD**, Nothing to disclose

